# Importance ranking and predictive model construction of WMSDs risk factors among shift-working nurses based on multiple machine-learning algorithms

**DOI:** 10.1186/s12912-026-04803-9

**Published:** 2026-05-27

**Authors:** Li-Chong Lai, Hai-Chen Wu, Xiao-Ying Cao, Yi-Fen Liao, Pin-Yue Tao, Xiao Pan, Shu-Yu Lu, Qi-Ni Pan, Dong-Mei Huang, Cai-Li Li, Peng-Xin Dong, Dong-Na Zhou, Hui-Qiao Huang

**Affiliations:** The Second Affiliated Hospital of Guangxi Medical University, No. 166 Daxue East Road, Xixiangtang District, Nanning City, Guangxi, 530007 China

**Keywords:** Nurse, Shift work, Work-related musculoskeletal disorders, Predictive model

## Abstract

**Background and objective:**

Nurses are essential for safeguarding public health, and their physical condition directly affects care quality and patient safety. Work-related musculoskeletal disorders (WMSDs) are highly prevalent in this workforce and may be amplified by circadian disruption. We therefore integrated individual and environmental risk factors, with special attention to night-shift characteristics, to identify and rank determinants of WMSDs among shift-working nurses. Seven machine-learning algorithms were compared to generate a comprehensive, validated prediction tool that enables managers and nurses to implement targeted, proactive interventions and reduce occupational injury.

**Methods:**

This study is a cross-sectional study. The general information, lifestyle, psychosocial data, working environment and shift characteristics of shift nurses were collected, and the influencing factors of WMSDs were analyzed. The mean square error increase and residual sum of squares are calculated, and the importance of influencing factors is sorted respectively. The independent influencing factors of WMSDs in shift nurses were included. After screening variables again by Lasso regression, seven prediction models of LDA, PLS, RDA, GLM, RF, SVM-Radial and SVM-Linear were established by machine learning. The AUC, accuracy and specificity median were used to evaluate the prediction efficiency, and the best prediction model was obtained and the accuracy of the prediction factors was verified.

**Results:**

Among 1 080 shift-working nurses, the WMSD prevalence at any body site was 85.19%. The top-ranked determinants were perceived control, perceived social support, Pittsburgh Sleep Quality Index (PSQI) score, chronotype, frequent bending over, night-shift nap duration, friend support, work support, family support, prolonged neck flexion and years in nursing. The LASSO-selected predictor set comprised dairy intake frequency, shift pattern, monthly night shifts, number of nurses on night duty, post-night-shift recovery days, post-night-shift catch-up sleep, frequent trunk flexion, prolonged neck flexion, PSQI, chronotype and perceived control. Random forest achieved the highest predictive performance (median AUC = 0.919).

**Conclusions:**

Individual characteristics, lifestyle, physical condition, occupational features, shift schedule, biomechanical load and psychosocial factors collectively influence WMSD occurrence in nurses. Random forest outperformed the other algorithms and should be carried out in conjunction with various factors in the model.

## Background

In the process of continuous improvement of the medical and health system, nurses, as an important force in ensuring the safety and physical health of the people, have received increasing attention to their work status and health level. “Shift-working nurses” are defined as registered nurses who rotate between at least two of the following duty periods: day, evening and night [1]. In the healthcare sector, 24-hour shift systems are mandatory to guarantee uninterrupted, high-quality nursing care. However, rotating between day, evening and night duties repeatedly desynchronizes the circadian clock, leading to suppressed nocturnal melatonin and altered reproductive-hormone profiles. This circadian disruption has been consistently associated with higher risks of breast and other cancers, cardiovascular events, metabolic syndrome and type 2 diabetes among shift-working nurses [2–4]. A comprehensive understanding of the determinants underlying these disorders and the implementation of evidence-based preventive interventions are therefore essential to safeguarding nurses’ occupational health and, by extension, maintaining high-quality patient care.

Work-related musculoskeletal disorders (WMSDs) are defined as discomfort, pain, numbness, or other symptoms occurring in one or more major body regions as a result of work or the work environment. These symptoms persist for ≥ 24 h and do not resolve with rest. A recent meta-analysis reported that the annual prevalence of WMSDs among Chinese nurses is 79%, with the lower back being the most frequently affected site, followed by the neck and shoulders [5]. This high prevalence and heavy burden have already precipitated attrition within the nursing workforce and a measurable decline in the quality of care delivered [6]. Over the past decade, the body of evidence on WMSD risk factors among nurses has expanded rapidly. In a cross-sectional survey of 1 803 nurses, Lin et al. [7] identified older age, longer tenure, specialty unit, ≥ 5 work-days per week and shift work as independent predictors. Mekonnen et al. [8]reported that academic degree, shift schedule and absence of health-and-safety training were significantly associated with low-back pain. Yao et al. [9]found that physical inactivity, night-shift rotation and negative affect collectively doubled WMSD risk. A multicentre study covering 20 tertiary ICUs in Hunan province revealed that female sex, unmarried status, high hazard-perception scores and poor ergonomic environments (e.g., lack of patient-lift devices or insufficient lifting space) markedly increased WMSD odds [10]. Taking a psychosocial perspective, Freimann et al. [11]demonstrated that permanent night duty and frequent emergency calls eroded resilience and job satisfaction, thereby amplifying musculoskeletal symptoms. Collectively, these findings indicate that WMSD determinants in nurses cluster into three domains: (i) basic demographics and lifestyle, (ii) work situation and biomechanical load, and (iii) psychosocial conditions.

Importantly, emerging evidence links WMSDs directly to circadian disruption. Skeletal muscle is the largest collection of peripheral circadian rhythms in the human body. Both the central and peripheral circadian rhythms regulate the interaction between the musculoskeletal system and energy metabolism. The skeletal muscle circadian rhythm is crucial in lipid and glucose metabolism, affecting the regulation of circadian rhythms in shift workers. A meta-analysis by Chang et al. [12]showed that shift-working nurses are significantly more likely to develop WMSDs than their fixed-day counterparts. Prolonged shift duration (> 9 h) is an independent predictor of WMSDs [13], and there is a clear dose–response relationship between years of shift work and disorder risk: every additional 5-year increment in shift tenure raises the prevalence of lower-limb WMSDs by 4% [14]. Moreover, a high frequency of night shifts amplifies the likelihood of multi-site pain [15]. Therefore, the present study collected detailed shift-related parameters—whether the nurse rotated shifts on a regular basis, lifetime night-shift tenure, number of night shifts per month, length of each night shift, duration of on-duty naps, and recovery days after night shifts—to quantify how circadian disruption influences WMSD occurrence.

In recent years, machine-learning techniques have gained prominence in artificial-intelligence-assisted diagnosis and prediction because of their speed, high precision, efficiency and security. Compared with conventional statistical approaches, machine-learning algorithms can simultaneously accommodate a large number of predictors, mitigate multicollinearity and automatically capture flexible, data-driven interactions, thereby improving both the accuracy and the efficiency of predictive models [16]. Therefore, our study takes shift-working nurses, a high-risk group characterized by circadian rhythm disorders, as the research subjects to enhance the clinical relevance of the research. Unlike previous studies that primarily focused on biomechanical factors, in addition to considering both individual characteristics and work-related biomechanical features, our study also pays attention to psychosocial factors and the specific circumstances of shift work, and ranks their importance. Furthermore, it employs seven different machine learning algorithms to validate the predictive performance of the model and the accuracy of the predictive variables, providing evidence-based grounds for the development of targeted prevention and intervention strategies for shift-working healthcare professionals.

## Subjects and methods

### Participants

Using convenience sampling method, nurses engaged in clinical work from 6 hospitals in 3 cities in Guangxi who meet the inclusion and exclusion criteria were selected as the research subjects, and an offline combined with online questionnaire survey was conducted. Inclusion criteria: (1) Holding a valid nursing license and having worked independently for ≥ 1 year; (2) Informed of the study aims and methods and willing to participate. Exclusion criteria: (1) Not currently on hospital duty (e.g., on study leave, maternity or sick leave); (2) Pregnant or breastfeeding within the past 12 months; (3) Pre-existing musculoskeletal conditions attributable to congenital spinal disorders, tumors, gynecological diseases or other non-occupational causes; (4) History of trauma or surgery within the past 12 months; (5) Musculoskeletal complaints clearly resulting from regular exercise, domestic overexertion or similar non-work factors. The study protocol was approved by the hospital ethics committee (Approval No. 2023-KY−0941).

Sample-size calculation: Using the formula N= deff[µα/^2^2p(1-p)]/δ^2^, with α=0.05, estimated prevalence *p* = 82.5% [17], the minimum required sample was 856. Allowing for 20% incomplete data, we set the final target at ≥ 1 027 shift-working nurses.

### Survey content

#### WMSD risk-factor questionnaire

1. Sociodemographics: age, sex, education, marital and parity status, BMI, waist and hip circumference, blood type.
2. Occupational exposures: department, professional title, length of service, shift pattern, monthly income, adequacy of workspace, frequent bending over, often carry heavy objects, maintain a lowered posture for a long time during work, work in an uncomfortable position frequently, bend down and turn around frequently during work, use of vibrating tools, physical/chemical hazards, exposure to cold draughts or temperature variations, perceived lighting quality, previous WMSD-prevention training.
3. Night-shift specifics: regular rotation, night shift working years, night shifts per month, length of each night shift, interval between two night shifts, night shift nap duration, number of nurses on night shift, recovery days after nights, duration of sleep after night shift, satisfaction with night-shift frequency.
4. Lifestyle and health: frequency of drinking tea/coffee/alcohol, frequency of consuming dairy products, daily water intake, second-hand smoke exposure, weekly exercise sessions, chronic disease history, self-rated health.

#### Work-related musculoskeletal disorders

The 9-item Nordic Musculoskeletal Questionnaire (NMQ-E) was used to assess pain or discomfort in nine body regions (neck, shoulder, upper back, lower back, elbow, wrist, hips/thighs, knee, ankles/feet) during the preceding 12 months [18]. Each region was coded 0 = no symptoms, 1 = symptoms present; participants reporting pain in ≥ 1 region were classified as WMSD-positive. Cronbach’s α for the scale in this study was 0.863.

#### Pittsburgh Sleep Quality Index (PSQI)

The 19-item PSQI evaluates seven components of sleep over the past month: subjective sleep quality, sleep latency, sleep duration, habitual sleep efficiency, sleep disturbances, use of sleep medication and daytime dysfunction [19]. Global scores range 0–21; >5 indicates poor sleep quality. Cronbach’s α= 0.763.

#### Morningness–eveningness questionnaire−5 (MEQ−5)

MEQ−5 is a simplified morning and night type scale, consisting of 5 questions with a score range of 4–25 [20]. The higher the score, the more inclined an individual’s circadian rhythm is towards the morning type, while the lower the score, the more inclined it is towards the night type. The Cronbach’s α of MEQ−5 in this study is 0.728.

#### Perceived social support scale (PSSS)

This scale consists of 12 items, including 3 dimensions: family support, friend support, and work support [21]. It uses a Likert 7-point scoring system, ranging from 1 (strongly disagree) to 7 (strongly agree), with higher scores indicating better social support. This study conducted reliability tests on the dimensions of job support, family support, friend support, and the overall scale of PSSS, which were 0.973, 0.944, 0.977, and 0.960, respectively.

#### Sense of agency scale (SoAS)

The 9-item Sense of Agency Scale developed by Tapal et al. [22], previously translated and validated in Chinese, was administered. Items are rated 1–7; items 2, 3, 6 and 7 are reverse-scored. Total scores range 9–63, with higher values reflecting stronger perceived control. Cronbach’s α= 0.822.

### Quality control

Before data collection, all research staff received standardized training on inclusion/exclusion criteria and operational definitions of every variable. On site, participants were given a uniform explanation of the study purpose, questionnaire completion instructions and estimated completion time; clarifications were provided when items were unclear. For online recruitment, the survey link and QR code were distributed through WeChat groups. The electronic questionnaire was programmed to allow only one response per device and to prevent submission until every item was answered. Each evening, newly received records were checked for completeness and logical consistency. After retrieval, data were double-entered and cross-verified. Records exhibiting patterned responses (e.g., identical answers across an entire page) or implausibly short completion times (< 5 min) were excluded.

### Statistical analysis

Descriptive statistics were generated with Excel and SPSS 23.0. Continuous variables with a normal distribution are presented as mean ± SD and were compared between groups using Student’s t-test or one-way ANOVA; non-normally distributed variables are expressed as median (inter-quartile range) and were compared with the Mann–Whitney U or Kruskal–Wallis test. Categorical data are summarized as frequencies and percentages, and group differences were tested with the χ² or Fisher’s exact test. Multivariable logistic regression was used to identify independent factors associated with WMSDs among shift-working nurses. All tests were two-tailed; *P* < 0.05 was considered statistically significant.

Variable importance was quantified with the randomForest package in R Studio (v4.3.3) using the increase in mean-squared error and residual sum of squares. Variables that were significant in univariate analyzes were entered as candidate predictors. Subsequent variable selection was performed with LASSO regression (glmnet package). Seven machine-learning algorithms—linear discriminant analysis (LDA), partial least squares (PLS), regularized discriminant analysis (RDA), generalized linear model (GLM), random forest (RF), radial-kernel support-vector machine (SVM-Radial) and linear-kernel SVM (SVM-Linear)—were implemented with the caret package. To obtain robust generalisability estimates and reduce evaluation bias introduced by different data-splitting schemes, we used repeated 10 × 10-fold cross-validation. The dataset was randomly divided into 10 approximately equal folds; in each of the 10 repeats, nine folds (90%) were used for training and the remaining fold (10%) for validation. The grid search with a tuning length of 5 was used for tuning parameters. Identical training and test folds were employed for all seven models. Model performance was assessed with the median area under the receiver-operating-characteristic curve (AUC), accuracy and specificity; the algorithm with the highest AUC was selected as the optimal predictor.

## Results

### Prevalence and single-factor analysis of WMSDs among shift nurses

A total of 1 080 shift-working nurses were included in our study, with a mean age of 30.29 ± 6.03 years. The prevalence of WMSDs in at least one body region was 85.19%. The prevalence by specific body region was as follows: neck 66.85%, shoulders 57.31%, upper back 39.44%, lower back 63.15%, elbows 33.33%, wrists 35.19%, hips/thighs 27.22%, knees 37.22%, and ankles/feet 35.83%. Univariate comparisons between nurses with and without WMSDs are summarized below.

#### Demographics, lifestyle and general health

Significant between-group differences (*P* < 0.05) were observed for age, blood type, second-hand smoke exposure, frequency of drinking tea/coffee, frequency of consuming dairy products, daily water intake, body weight, waist circumference, hip circumference, self-reported hyperlipidaemia, chronic bronchitis or other respiratory diseases, gastro-intestinal disorders, and self-rated health status (Table 1).

**Table 1:** Analysis of WMSDs of demographic data, lifestyle and physical health of shift nurses

Variables		WMSDs in the past year		t/χ^2^/Z	*P*
		Yes(*n* = 920)	No(*n* = 160)		
Age	30.29 ± 6.03	30.47 ± 6.07	29.26 ± 5.76	2.345	0.019
Sex				1.700	0.192
Male	89(8.24)	80(89.89)	9(10.11)		
Female	991(91.76)	840(84.76)	151(15.24)		
Education				1.261	0.532
Junior college or below	295(27.31)	247(83.73)	48(16.27)		
Bachelor	771(71.39)	662(85.86)	109(14.14)		
Master or above	14(1.30)	11(78.57)	3(21.43)		
Marital status				0.702	0.402
Married	534(49.44)	450(84.27)	84(15.73)		
Unmarried / divorced / widowed	546(50.56)	470(86.08)	76(13.92)		
Number of children				2.452	0.484
0	607(56.20)	522(86.00)	85(14.00)		
1	214(19.81)	182(85.05)	32(14.95)		
2	245(22.69)	202(82.45)	43(17.55)		
>2	14(1.30)	14(100.00)	0(0.00)		
Blood type				12.896	0.005
A	252(23.33)	220(87.30)	32(12.70)		
B	325(30.09)	279(85.85)	46(14.15)		
AB	102(9.44)	96(94.12)	6(5.88)		
O	401(37.13)	325(81.05)	76(18.95)		
Weekly exercise frequency				1.987	0.370
0	535(49.54)	457(85.42)	78(14.58)		
1–3	454(42.04)	390(85.90)	64(14.10)		
>3	91(8.43)	73(80.22)	18(19.78)		
Second-hand smoke exposure				20.538	<0.001
Yes	357(33.06)	329(92.16)	28(7.84)		
No	723(66.94)	591(81.74)	132(18.26)		
frequency of drinking tea/coffee				9.773	0.021
≥1 time per day	201(18.61)	177(88.06)	24(11.94)		
1–6 times per week	375(34.72)	329(87.73)	46(12.27)		
1–3 times per month	134(12.41)	116(86.57)	18(13.43)		
Rarely	370(34.26)	298(80.54)	72(19.46)		
Dairy-product intake				21.875	<0.001
≥1 time per day	320(29.63)	248(77.50)	72(22.50)		
1–6 times per week	504(46.67)	444(88.10)	60(11.90)		
1–3 times per month	147(13.61)	129(87.76)	18(12.24)		
Rarely	109(10.09)	99(90.83)	10(9.17)		
Daily water intake				42.646	<0.001
<500 mL	171(15.83)	150(87.72)	21(12.28)		
500–999 mL	399(36.94)	335(83.96)	64(16.04)		
1000–1499 mL	285(26.39)	255(89.47)	30(10.53)		
1500–2000 mL	159(14.72)	141(88.68)	18(11.32)		
>2000 mL	66(6.11)	39(59.09)	27(40.91)		
Body weight	54.54 ± 8.69	54.83 ± 8.96	52.93± 6.75	11.892	0.002
Waist circumference	71.33 ± 8.07	71.66 ± 8.27	69.49± 6.59	5.630	<0.001
Hip circumference	90.44 ± 9.99	90.83 ± 9.97	88.24± 9.81	0.107	0.002
Hyperlipidaemia				6.422	0.011
Yes	1001(92.69)	845(84.42)	156(15.58)		
No	79(7.31)	75(94.94)	4(5.06)		
Chronic bronchitis or other respiratory diseases				8.566	0.003
Yes	999(92.50)	842(84.28)	157(15.72)		
No	81(7.50)	78(96.30)	3(3.70)		
Gastroenteritis or other digestive disorders				3.538	0.048
Yes	959(88.80)	810(84.46)	149(15.54)		
No	121(11.20)	110(90.91)	11(9.09)		
Self-rated health status				96.444	<0.001
Excellent	145(13.43)	90(62.07)	55(37.93)		
Good	349(32.31)	284(81.38)	65(18.62)		
Fair	486(45.00)	449(92.39)	37(7.61)		
Poor	100(9.26)	97(97.00)	3(3.00)		
PSQI	6.88± 3.67	7.34 ± 3.56	4.24 ± 3.11	10.439	<0.001
Subjective sleep quality	1.34± 0.74	1.41 ± 0.72	0.95 ± 0.76	−7.466	<0.001
Sleep latency	1.67± 0.96	1.74 ± 0.94	1.26 ± 0.98	−6.00	<0.001
Sleep duration	1.33± 0.84	1.43 ± 0.83	0.76 ± 0.69	−9.588	<0.001
Sleep efficiency	0.61± 0.86	0.65 ± 0.88	0.39 ± 0.66	−3.462	<0.001
Sleep disturbances	1.13± 0.66	1.19 ± 0.63	0.83 ± 0.73	−6.593	<0.001
Hypnotic medication use	0(0, 0)	0(0, 0)	0(0, 0)	−3.208	<0.001
Daytime dysfunction	1.58± 0.99	1.69 ± 0.96	0.94 ± 0.91	−9.153	<0.001
Chronotype	13.46± 3.72	13.06 ± 3.53	15.80 ± 3.92	−8.927	<0.001
Social support	60.63± 16.97	59.51 ± 16.69	66.66 ± 15.66	−5.045	<0.001
Work support	18.87± 7.05	18.45 ± 6.94	21.24 ± 7.26	4.650	<0.001
Family support	21.44± 5.78	21.22 ± 5.81	22.73 ± 5.41	3.052	0.002
Friend support	20.32± 6.53	19.73 ± 6.61	23.69 ± 4.83	7.248	<0.001
Sense of agency	46.66± 7.73	45.58 ± 8.07	51.06 ± 5.985	−8.196	<0.001

#### Occupational and shift characteristics

Univariate analysis revealed statistically significant differences (*P* < 0.05) between nurses with and without WMSDs in the following variables: nursing tenure (years), intention to choose nursing profession, clinical department, shift-rotation pattern, lifetime night-shift tenure, regularity of shift rotation, number of night shifts per month, duration of each night shift, interval between consecutive night duties, on-duty nap length during nights, number of nurses on night duty, patient load on day shifts, post-night-shift recovery days, catch-up sleep after night blocks, and satisfaction with night-shift frequency (Table 2).

**Table 2:** Single factor analysis of WMSDs annual occurrence of work and shift characteristics of shift nurses

Variables		WMSDs in the past year			t/χ^2^/Z	*P*
		Yes(*n* = 920)	No(*n* = 160)			
Length of service	8.63 ± 6.24	8.81 ± 6.29		7.58 ± 5.871	2.300	0.022
Intention to choose nursing profession					16.904	<0.001
Self-selected	609(56.39)	495(81.28)		114(18.72)		
Recommended by others	415(38.46)	374(90.12)		41(9.88)		
Reassigned	56(5.19)	51(91.07)		5(8.93)		
Department					106.583	<0.001
Internal medicine	341(31.57)	306(89.74)		35(10.26)		
Surgery	298(27.59)	270(90.60)		28(9.40)		
ICU	120(11.11)	104(86.67)		16(13.33)		
Emergency	79(7.31)	54(68.35)		25(31.65)		
Pediatrics	92(8.52)	50(54.35)		42(45.65)		
Obstetrics & gynecology	89(8.24)	78(87.64)		11(12.36)		
Operating room	61(5.65)	58(95.08)		3(4.92)		
Professional title					2.723	0.436
Staff nurse	281(26.02)	237(84.34)		44(15.66)		
Senior nurse	464(42.96)	402(86.64)		62(13.36)		
Supervisor nurse	307(28.43)	255(83.06)		52(16.94)		
Associate chief nurse or above	28(2.59)	26(92.86)		2(7.14)		
Average monthly income					7.018	0.071
< 4 000	106(9.81)	91(85.85)		15(14.15)		
4 000–6 000	467(43.24)	410(87.79)		57(12.21)		
6 001–9 000	376(34.81)	306(81.38)		70(18.62)		
> 9 000	131(12.13)	113(86.26)		18(13.74)		
Shift pattern					6.103	0.013
Day-Night (DN)	517(47.87)	426(82.40)		91(17.60)		
Morning-Evening-Night (APN)	563(52.13)	494(87.74)		69(12.26)		
History of work error					3.819	0.051
Yes	536(49.63)	468(87.31)		68(12.69)		
No	544(50.37	452(83.09)		92(16.91)		
Years of shift work	8.34± 6.14	8.52± 6.20		7.28± 0.13	2.358	0.019
Regular shift rotation					8.400	0.004
Yes	805(74.54)	671(83.35)		134(16.65)		
No	275(25.46)	249(90.55)		26(9.45)		
Night shifts per month					44.480	<0.001
≤ 3	159(14.72)	110(69.18)		49(30.82)		
4–5	595(55.09)	510(85.71)		85(14.29)		
≥ 6	326(30.19)	300(92.02)		26(7.98)		
Length of each night shift					4.753	0.029
< 9 h	253(32.69)	207(81.82)		46(18.18)		
9–12 h	503(46.57)	427(84.89)		76(15.11)		
> 12 h	324(30.00)	286(88.27)		38(11.73)		
Interval between night shifts					17.638	<0.001
< 2 days	142(13.15)	123(86.62)		19(13.38)		
2–3 days	262(24.26)	241(91.98)		21(8.02)		
4–7 days	554(51.30)	462(83.39)		92(16.61)		
> 7 days	122(11.30)	94(77.05)		28(22.95)		
Night shift nap duration					56.553	<0.001
0 h	332(30.74)	285(85.84)		47(14.16)		
1 h	114(10.56)	111(97.37)		3(2.63)		
2 h	368(34.07)	332(90.22)		36(9.78)		
≥ 3 h	266(24.63)	192(72.18)		74(27.82)		
Average number of nurses on duty per night shift					22.516	<0.001
1	244(22.59)	220(90.16)		24(9.84)		
2	540(50.00)	472(87.41)		68(12.59)		
≥ 3	296(27.41)	228(77.03)		68(22.97)		
Average number of patients assigned per day shift					39.927	<0.001
≤ 2	41(3.80)	21(51.22)		20(48.78)		
3–5	133(12.31)	115(86.47)		18(13.53)		
6–8	212(19.63)	179(84.43)		33(15.57)		
> 8	694(64.26)	609(87.75)		89(12.82)		
Post-night-shift recovery days					25.960	<0.001
1 day	155(14.35)	142(91.61)		13(8.39)		
2 days	783(72.50)	676(86.33)		107(13.67)		
> 2 days	142(13.15)	102(71.83)		40(28.17)		
Sleep duration after night shift					60.426	<0.001
< 4 h	307(28.43)	289(94.14)		18(5.86)		
4–6 h	567(52.50)	488(86.07)		79(13.93)		
> 6 h	206(19.07)	143(69.42)		63(30.58)		
Satisfaction with night-shift frequency					46.777	<0.001
Satisfied	263(24.35)	197(74.90)		66(25.10)		
Neutral	613(56.76)	524(85.48)		89(14.52)		
Dissatisfied	204(18.89)	199(97.55)		5(2.45)		

#### Occupational biomechanical factors

Univariate comparisons showed significant between-group differences (*P* < 0.05) for the following biomechanical variables: frequent bending over, regular heavy lifting, prolonged neck flexion, working in awkward postures, simultaneous trunk flexion and rotation, use of vibrating tools, exposure to physical hazards, exposure to chemical hazards, working in cold draughts or variable ambient temperatures, perceived lighting quality, and attendance at WMSD-prevention training (Table 3).

**Table 3:** Single factor analysis of WMSDs annual occurrence of work biomechanics of shift nurses

Variables		WMSDs in the past year			t/χ^2^/Z	*P*
		Yes(*n* = 920)		No(*n* = 160)		
Adequacy of workspace					2.438	0.118
Yes	782(72.41)	658(84.14)	124(15.86)			
No	298(27.59)	262(87.92)	36(12.08)			
frequent bending over					131.167	<0.001
Yes	866(80.19)	791(91.34)	75(8.66)			
No	214(19.81)	129(60.28)	85(39.72)			
Regular heavy lifting					29.057	<0.001
Yes	596(55.19)	539(90.44)	57(9.56)			
No	484(44.81)	381(78.72)	103(21.28)			
Prolonged neck flexion during work					102.568	<0.001
Yes	731(67.69)	678(92.75)	53(7.25)			
No	349(32.31)	242(69.34)	107(30.66)			
Frequent awkward postures					103.497	<0.001
Yes	607(56.20)	576(94.89)	31(5.11)			
No	473(43.80)	344(72.73)	129(27.27)			
Combined trunk flexion and rotation					75.515	<0.001
Yes	664(61.48)	615(92.62)	49(7.38)			
No	416(38.52)	305(73.32)	111(26.68)			
Use of vibrating tools					21.746	<0.001
Yes	223(20.65)	212(95.07)	11(4.93)			
No	857(79.35)	708(82.61)	149(17.39)			
Exposure to physical hazards					49.221	<0.001
Rarely	429(39.72)	345(80.42)	84(19.58)			
1–4 times per month	324(30.00)	259(79.94)	65(20.06)			
2–6 times per week	174(16.11)	166(95.40)	8(4.60)			
1–3 times per day	92(8.52)	90(97.83)	2(2.17)			
> 3 times per day	61(5.65)	60(98.36)	1(1.64)			
Exposure to chemical hazards					18.793	<0.001
Rarely	587(54.35)	483(82.28)	104(17.72)			
1–4 times per month	244(22.59)	204(83.61)	40(16.39)			
2–6 times per week	108(10.00)	102(94.44)	6(5.56)			
1–3 times per day	75(6.94)	71(94.67)	4(5.33)			
> 3 times per day	66(6.11)	60(90.91)	6(9.09)			
Cold draughts or temperature variations in work area					42.785	<0.001
Yes	493(45.65)	458(92.90)	35(7.10)			
No	587(54.35)	462(78.71)	125(21.29)			
Perceived lighting quality at workstation					8.997	0.011
Too bright	197(18.24)	156(79.19)	41(20.81)			
Comfortable	733(67.87)	640(87.31)	93(12.69)			
Too dim	150(13.89)	124(82.67)	26(17.33)			
Attendance at WMSD-prevention training					9.413	0.002
Yes	261(24.17)	207(79.31)	54(20.69)			
No	819(75.83)	713(87.06)	106(12.94)			

### Multivariable analysis of annual WMSD occurrence

Using the presence or absence of any WMSD during the preceding 12 months as the dependent variable, we entered the 53 statistically significant factors identified in univariate analysis as candidate predictors. Multivariable logistic regression retained 18 independent determinants (*P* < 0.05): dairy-product intake frequency, daily water intake, intention to choose nursing profession, clinical department, shift pattern, regular shift rotation, number of night shifts per month, number of nurses on night duty, post-night-shift recovery days, catch-up sleep after nights, frequent bending over, prolonged neck flexion, use of vibrating tools, waist circumference, sleep-onset time, PSQI score, chronotype and sense of agency. Likelihood-ratio χ² = 373.384, *P* < 0.001, indicating adequate model fit (Table 4).

**Table 4:** Multivariate analysis of WMSDs in shift nurses

	B	SE	Wald X^2^	*P*	OR	95% CI
Frequency of dairy-product intake	0.364	0.132	7.591	0.006	1.439	1.111–1.865
Daily water intake	0.328	0.114	8.321	0.004	1.389	1.111–1.736
Route into nursing	0.64	0.215	8.848	0.003	1.897	1.244–2.892
Clinical department	-0.138	0.062	5.032	0.025	0.871	0.772–0.983
Shift-rotation pattern	1.045	0.24	18.894	<0.001	2.843	1.775–4.555
Regular shift rotation	0.627	0.297	4.463	0.035	1.872	1.046–3.349
Night shifts per month	0.724	0.189	14.733	<0.001	2.063	1.425–2.986
Number of nurses on night duty	-0.507	0.17	8.876	0.003	0.602	0.431–0.841
Post-night-shift recovery days	-0.701	0.219	10.265	0.001	0.496	0.323–0.762
Catch-up sleep after nights	-0.602	0.179	11.258	0.001	0.548	0.385–0.779
frequent bending over	0.672	0.292	5.292	0.021	1.959	1.105–3.473
Prolonged neck flexion	0.949	0.272	12.154	<0.001	2.582	1.515–4.401
Use of vibrating tools	1.168	0.408	8.205	0.004	3.217	1.446–7.156
Waist circumference	0.037	0.015	6.243	0.012	1.038	1.008–1.069
Sleep-onset time	-0.631	0.187	11.339	0.001	0.532	0.369–0.768
PSQI global score	0.288	0.06	22.976	<0.001	1.334	1.186–1.501
Chronotype	-0.207	0.042	24.271	<0.001	0.813	0.749–0.883
Sense of agency	-0.087	0.019	21.816	<0.001	0.917	0.884–0.951
Constant	2.81	1.772	2.515	0.113	16.615	

### Importance ranking of factors associated with annual WMSD occurrence

**Fig. 1:**
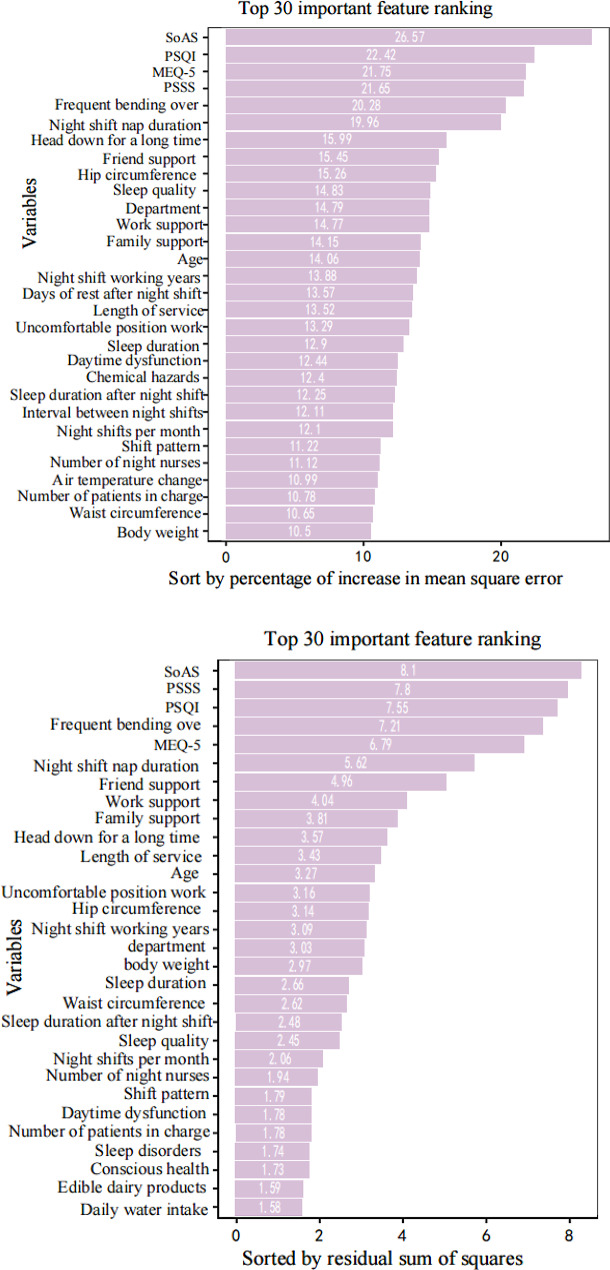
The importance ranking of influencing factors of nurses’ WMSDs based on random forest regression model analysis

We ranked the 53 candidate predictors by variable importance using both the increase in mean-squared error (MSE) and the residual sum of squares (RSS); the resulting rankings are displayed in Fig. 1. Regardless of which metric was used, the top determinants of WMSDs among shift-working nurses were consistently: sense of agency, perceived social support, PSQI global score, chronotype, frequent bending over, on-duty nap duration during night shifts, friend support, work support, family support, long term bowing and years of work experience.

### Model development and validation

#### Final predictor selection with LASSO regression

**Fig. 2:**
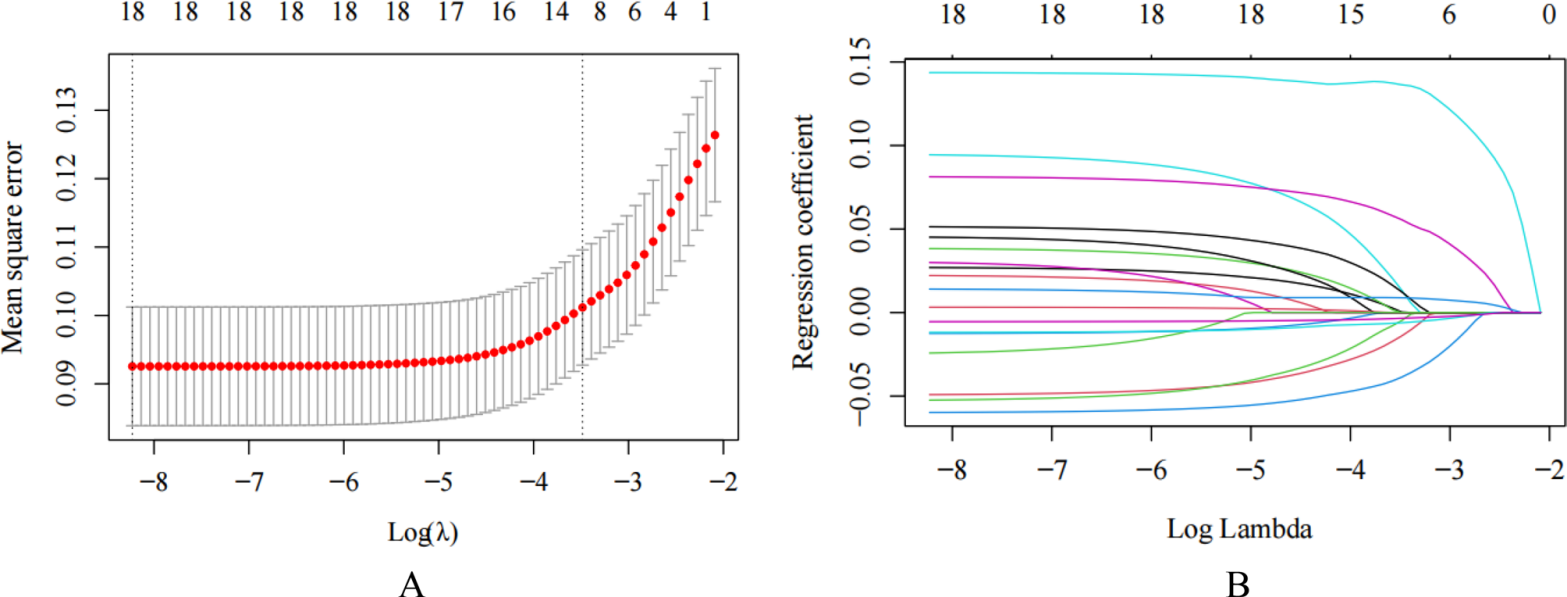
Lasso regression model to screen predictive variables. **A**: Lasso coefficient distribution for screening feature variables; **B**: Lasso model adaptive method for optimal λ value selection

The 18 variables retained in the multivariable logistic model were entered into a LASSO regression to further reduce over-fitting and select the most parsimonious set. Ten-fold cross-validation identified the optimal tuning parameter (λ = 0.03067). Eleven predictors with non-zero coefficients were retained: dairy-product intake frequency, shift-rotation pattern, number of night shifts per month, number of nurses on night duty, post-night-shift recovery days, catch-up sleep after nights, frequent trunk flexion, prolonged neck flexion, PSQI global score, chronotype and sense of agency (Fig. 2). Variable coding is shown in Table 5.

A B.

**Table 5:** Variable assignment method

	Variables	Assignment method
1	Dairy-product intake	≥ 1 time per day= 1, 1–6 times per week= 2, 1–3 times per month= 3, Rarely= 4
2	Shift pattern	DN = 1, APN = 2
3	Night shifts per month	≤ 3 = 1,4–5 = 2, ≥ 6 = 3
4	Number of night nurses	1 person = 1,2 person = 2; ≥ 3 people = 3
5	Night shift days off	1 day = 1, 2 days = 2; > 2 days = 3
6	Duration of sleep after night shift	6 h = 3
7	He often bends heavily	Yes = 1, No = 0
8	Head down for a long time	Continuous variables
9	PSQI	Continuous variable
10	MEQ-5	Continuous variable
11	SoAS	Continuous variable

#### Model construction and performance comparison

**Fig. 3:**
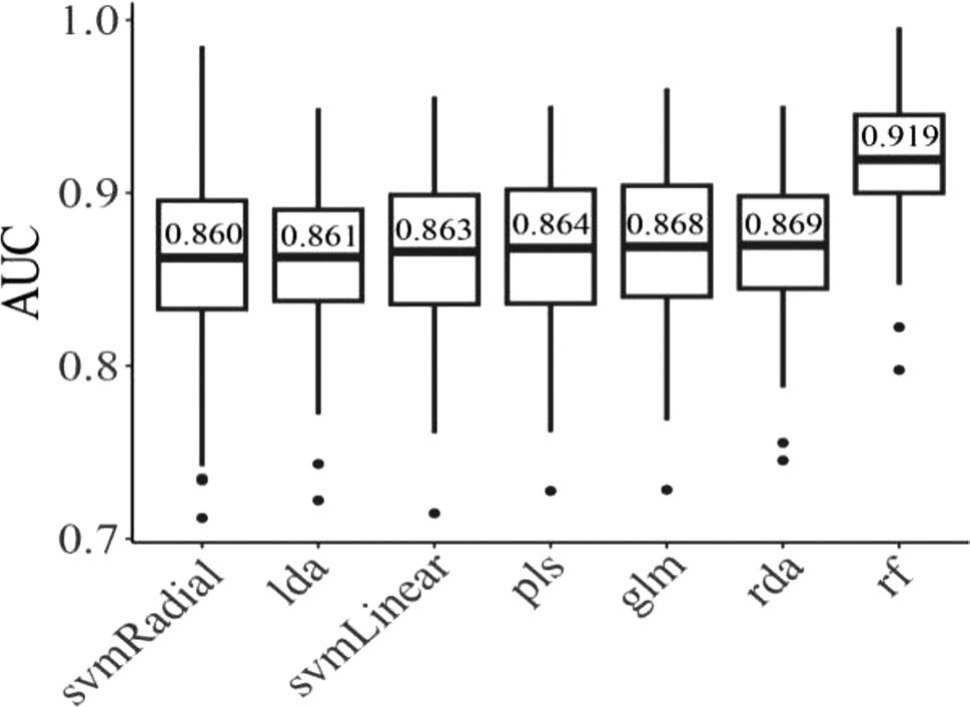
Comparison of AUC medians of prediction models constructed by 7 machine learning algorithms

Seven algorithms (LDA, PLS, RDA, GLM, RF, SVM-Radial and SVM-Linear) were trained on the 11 LASSO-selected features. There is no significant difference in the training time of each model. After 100 repetitions of stratified 10-fold cross-validation on the full dataset, median AUCs are displayed in Fig. 3; corresponding accuracy and specificity are summarized in Table 6. All seven models achieved good discriminative ability, confirming the predictive value of the 11-feature set. Random forest delivered the highest performance, with a median AUC of 0.919, and was therefore retained as the final prediction tool.

**Table 6:** Performance comparison of prediction models constructed by seven machine learning algorithms

Model	AUC	specificity	accuracy
rf	0.919(0.899–0.945)	0.981(0.978–0.989)	0.923(0.907–0.935)
glm	0.868(0.840–0.904)	0.973(0.967–0.978)	0.901(0.889–0.917)
pls	0.864(0.836–0.902)	0.995(0.989-1.000)	0.884(0.870–0.889)
rda	0.869(0.845–0.898)	0.954(0.934–0.967)	0.885(0.870–0.907)
svmLinear	0.863(0.836–0.899)	0.978(0.967–0.989)	0.895(0.887–0.907)
lda	0.861(0.837–0.890)	0.958(0.945–0.978)	0.884(0.870–0.898)
svmRadial	0.860(0.833–0.896)	0.979(0.967–0.989)	0.905(0.889–0.917)

Note: Each algorithm was evaluated over 100 cross-validation runs; values are reported as median (inter-quartile range)

## Discussion

### Importance ranking of determinants of WMSDs in shift-working nurses

The influencing factors of WMSDs related to shift nursing work have multidimensional mechanisms, and their importance is ranked in the following order: (i) sense of agency, (ii) perceived social support, (iii) PSQI score, (iv) chronotype, (v) frequent bending over and (iv) night shift nap duration. Sense of agency emerged as the most important predictor. Nurses who perceive high control over work pace and task allocation can autonomously adjust working postures and redistribute physical loads, thereby lowering musculoskeletal strain [23]. Emotional and instrumental support from colleagues, supervisors and family can significantly alleviate occupational stress, thereby indirectly reducing compensatory muscle recruitment [24]. Previous studies have shown that nurses who perceive low social support report a higher frequency of neck-and-shoulder pain; this is presumably because diminished psychological stress translates into lower resting muscle tension [25]. PSQI is a quantitative indicator of fatigue accumulation, and poor sleep quality directly leads to circadian rhythm disorders and growth hormone secretion inhibition, weakening muscle repair ability. Fragmented sleep caused by frequent night shifts is more likely to trigger compensatory strength in the elbow [26]. Meanwhile, deep sleep is crucial for reducing physical fatigue, but shift workers’ daytime sleep duration conflicts with their body’s sleep wake cycle, often making it difficult to fall asleep, maintain sleep, or sleep calmly. Deep sleep is significantly reduced, making it difficult to feel rested after sleep, thereby exacerbating muscle fatigue [12]. In addition, insufficient sleep can increase pain sensitivity after acute muscle soreness and increase the incidence of WMSDs [27]. The type of circadian rhythm can reflect differences in physiological adaptability, and the morning or evening circadian rhythm affects nurses’ tolerance to shifts [28]。Evidence indicates that distinct sleep-timing phenotypes and divergent sleep-quality scores independently shape both physical exhaustion and psychological burnout. Evening-type nurses, whose endogenous rhythms are phase-delayed, maintain a relatively stable blood-pressure pattern across APN rotations. Nevertheless, chronic shift work still intensifies sleep fragmentation in this group, suppressing nocturnal growth-hormone secretion and blunting overnight muscle repair [29]. 。Frequent bending over is a direct cause of mechanical injury. Employees who frequently bend over to operate the lumbar intervertebral disc will experience an increase in peak pressure, leading to interlaminar shear and fatigue microcracks in the fibrous ring, accelerating intervertebral disc degeneration [30]. Night-shift nap duration represents a critical short-term recovery window. Brief on-duty naps can transiently restore attention [31]. The observed ranking unveils a psycho-physiological-behavioral interplay underlying WMSDs. The top three determinants (sense of agency, social support and sleep quality) are amenable to managerial interventions such as flexible scheduling and team-based collaboration training, whereas the latter three require ergonomic redesign coupled with individual behavioral counseling. Future research can focus on the matching degree between circadian rhythm types and shift patterns to develop precise protective strategies.

### Predictor profile of WMSDs among shift-working nurses

#### Personal-lifestyle domain

The development of WMSDs in shift-working nurses is closely linked to personal lifestyle. First, dairy-product intake frequency serves as a proxy for calcium-homeostasis status: low-frequency consumption reduces intestinal calcium absorption efficiency, thereby impairing skeletal-muscle excitation–contraction coupling and destabilizing bone-mineral density [31]. Calcium imbalance accelerates lumbar-disc degeneration, a risk amplified in night-shift nurses whose irregular meal patterns favor insufficient calcium intake [32]. Daily water intake governs muscle-metabolite clearance. Inadequate hydration slows the removal of catabolites such as lactate, promoting fatigue accumulation and low-grade intramuscular inflammation [33]. Moreover, dehydration diminishes synovial fluid secretion within the carpal tunnel, facilitating the onset of carpal-tunnel syndrome among nurses performing repetitive wrist movements [34]. Waist circumference is closely linked to metabolic inflammation. Adult abdominal obesity indicates visceral fat accumulation; pro-inflammatory cytokines such as IL-6 and TNF-α released from visceral adipose tissue enter the circulation and stimulate skeletal-muscle fibrosis, thereby increasing the incidence of chronic lumbar-strain injuries [35]. Sleep-onset time reflects circadian phase alignment. Nurses who fall asleep after midnight exhibit a delayed nocturnal-melatonin peak, shortening the duration of deep sleep and compressing the critical window for muscle repair [36]. The PSQI quantifies fatigue accumulation. Higher global PSQI scores predict faster declines in muscular endurance, because reduced slow-wave sleep suppresses ultrastructural myofibre restoration [37]. When both difficulty initiating sleep and early-morning awakening are present, the frequency of neck-and-shoulder pain increases 2.3-fold [38].

#### Occupational and shift-related characteristics

The development of WMSDs in shift-working nurses is intimately linked to job-design and rostering factors. First, career-choice motivation matters: nurses who entered the profession with low intrinsic identification exhibit markedly higher burnout rates [39]. Because their tolerance for repetitive manual tasks is reduced, these individuals appear to amplify musculoskeletal strain through sustained psychophysiological stress pathways that increase resting muscle tension [40]. Second, years of tenure and compensatory capacity play a role. Although long-term nursing workers have improved operational efficiency due to accumulated experience, previous studies have shown that their prevalence of WMSDs is actually higher, suggesting the cumulative effect of chronic injuries [41]. This paradox is likely mediated by age-related declines in muscle-repair capacity and the persistence of previously unresolved micro-injuries that reduce the effectiveness of compensatory movement patterns [42]. Finally, roster design and night-shift workload are critical. High night-shift density, insufficient catch-up sleep after nights, and fewer recovery days each independently increase WMSD risk, largely via circadian disruption and progressive muscle-fatigue accumulation [43]. Inadequate night staffing forces individual nurses to perform multiple, physically demanding tasks without assistance, heightening both psychological strain and biomechanical loading [44]. Moreover, compressed rosters that shorten inter-shift intervals prevent full muscular recovery and further amplify musculoskeletal injury [45]. Therefore, when implementing APN continuous rosters, hospitals should strictly enforce adequate rest intervals between night shifts, guarantee sufficient post-night recovery days and catch-up sleep duration to attenuate muscle fatigue, and provide intensive biomechanics training during orientation to establish protective movement patterns that minimize cumulative musculoskeletal strain [46].

#### Occupational biomechanical exposures

Prolonged neck flexion is a ubiquitous precipitant of WMSDs in nurses. Tasks such as drug preparation, intravenous insertion and electronic charting oblige staff to maintain a head-down posture for extended periods, continuously loading the cervical and periscapular muscles and precipitating myofascial fatigue and micro-injury [47]. Chronic compression of the posterior disc compartments also accelerates cervical-spondylotic change, further compromising musculoskeletal health [48]. Exposure to vibrating tools, though less frequent, constitutes an additional hazard. In departments such as neurology or rehabilitation, nurses may operate or assist with vibration-based therapeutic devices; transmitted oscillations increase forearm and hand muscular loading and have been associated with carpal-tunnel and elbow-tendon pathologies [49]. Suggest implementing posture management and health intervention programs: nurses should be prompted to adjust working posture at regular intervals, with provision of brief, scheduled neck-relief breaks, while mandatory, progressive exercise sessions emphasizing core-muscle strengthening are introduced to enhance segmental stability and mitigate cervical load.

### Comparison of machine-learning models for predicting WMSDs in shift-working nurses

Using the median AUC across repeated cross-validation folds attenuates over-fitting and provides a more conservative estimate of generalisability [50]. Therefore, we adopted median AUC as the primary metric for model comparison. After 100 repetitions of stratified 10-fold cross-validation, the random-forest (RF) algorithm achieved the highest median AUC (0.919) among the seven candidate models, corroborating the validity of the 11 LASSO-selected predictors and offering a practical decision-support tool for mitigating shift-work-related musculoskeletal morbidity. RF aggregates an ensemble of classification trees by bootstrapping both observations and features, enabling robust handling of high-dimensional, heterogeneous data [51]. In the present context—where WMSD risk is jointly shaped by lifestyle habits, shift schedules, workload and ergonomic exposures—RF efficiently captures complex, non-linear interactions among these domains while remaining resilient to outliers, multicollinearity and class imbalance [52]. Consequently, RF provides a reliable and readily deployable predictive framework for occupational health teams seeking to target high-risk shift-working nurses with tailored interventions.

However, several limitations should be acknowledged. First, the cross-sectional design, while effective in revealing the current status of WMSDs and associated factors among shift-working nurses, precludes the establishment of causal relationships between variables. Second, defining WMSDs as the presence of symptoms in at least one body region may have led to an inflated prevalence estimate. Finally, this study only conducted internal validation and lacked independent external samples to further test the performance of the model. The generalization ability of the model in heterogeneous populations is still unclear. Future research should consider employing a prospective cohort design to elucidate the temporal sequence and dynamic evolution of the relationships between various factors and WMSD onset among shift-working nurses. Additionally, subsequent studies could refine the ascertainment of WMSDs by incorporating multidimensional indicators, including symptom severity, functional impairment, and clinical diagnosis, to enhance the precision of outcome definition.

## Conclusion

Previous research has focused predominantly on ergonomic stressors, largely overlooking how shift schedules per se influence WMSD risk among nurses. By simultaneously evaluating lifestyle, shift-related and biomechanical exposures, and by benchmarking seven machine-learning algorithms, the present study delivers an evidence-based, multi-factorial framework for safeguarding nursing workforce health. Finally, the current investigation is cross-sectional, although it delineates the prevalence of WMSDs and their association with shift patterns, causal inference cannot be drawn. Future research may consider adopting a longitudinal design to dynamically evaluate the impact of shift cycles and speeds on the occurrence and development of WMSDs, and conduct comprehensive interdisciplinary applied research.

## Data Availability

The datasets used and/or analyzed in the present study are available from the corresponding author on reasonable request.

## Abbreviations

WMSDs: Work-related musculoskeletal disorders
PSQI: Pittsburgh Sleep Quality Index
NMQ-E: Nordic Musculoskeletal Questionnaire
MEQ-5: Morningness–Eveningness Questionnaire-5
PSSS: Perceived Social Support Scale
SoAS: Sense of Agency Scale
LDA: Linear discriminant analysis
PLS: Partial least squares
RDA: Regularized discriminant analysis
GLM: Generalized linear model
RF: Random forest
SVM-Radial: Radial-kernel support-vector machine
SVM-Linear: Linear-kernel SVM
MSE: Mean-squared error
RSS: Residual sum of squares
RF: Random-forest
